# *Echinococcus multilocularis* (Cestoda, Cyclophyllidea, Taeniidae): origin, differentiation and functional ultrastructure of the oncospheral tegument and hook region membrane

**DOI:** 10.1007/s00436-018-5752-7

**Published:** 2018-01-15

**Authors:** Zdzisław Świderski, Jordi Miquel, Samira Azzouz-Maache, Anne-Françoise Pétavy

**Affiliations:** 1 Witold Stefański Institute of Parasitology, Polish Academy of Sciences, 51/55 Twarda Street, 00-818 Warszawa, Poland; 2 Secció de Parasitologia, Departament de Biologia, Sanitat i Medi Ambient, Facultat de Farmàcia i Ciències de l’Alimentació, Universitat de Barcelona, Av. Joan XXIII, sn, 08028 Barcelona, Spain; 3 Institut de Recerca de la Biodiversitat (IRBio), Facultat de Biologia, Universitat de Barcelona, Av. Diagonal, 645, 08028 Barcelona, Spain; 4 Laboratoire de Parasitologie et Mycologie Médicale, Faculté de Pharmacie, Université Claude Bernard-Lyon 1, 8 Av. Rockefeller, 69373 Lyon Cedex 08, France

**Keywords:** *Echinococcus multilocularis*, Taeniid cestodes, Preoncospheral differentiation, Oncospheral tegument, Morphogenesis of hook region membrane, Oncoblasts, Oncospheral hooks, Ultrastructure

## Abstract

Both the oncospheral tegument and the hook region membrane (HRM) of *Echinococcus multilocularis* hexacanths originate from a syncytial binucleate complex that appears in the early stage of morphogenesis and organogenesis of the hexacanth larva. The primordium of this binucleate complex forms a binucleate syncytial cap or “calotte” situated beneath the inner envelope at one pole of the developing embryo. During oncospheral differentiation, the binucleate perikaryon of the syncytial cap is sunk progressively deeper into the central part of the embryo, but remains always connected with the distal cytoplasm by a tendrillar cytoplasmic connection or bridge. Following migration or sinking of the binucleate perikaryon, numerous cytoplasmic vesicles appear in the distal cytoplasm. These vesicles fuse progressively together and form a single large cavity or lacuna. The walls of this cavity are becoming at this point the walls of two delaminated layers: (1) the distal anucleated cytoplasmic layer is transformed into the oncospheral tegument and (2) the proximal thin cytoplasmic layer is transformed into the “hook region membrane”. This delamination of the initially compact layer of distal cytoplasm into two layers seems to be closely associated with differentiation of oncospheral hooks, the elongating blades of which protrude progressively into a newly formed cavity. The pressure of hook blades on the hook region membrane appears to facilitate its further separation from the basal layer of distal cytoplasm which is transformed into the peripheral layer of oncospheral tegument. In the mature oncosphere, the surface of this peripheral layer forms a regular brush border of cytoplasmic processes or microvilli and represents the true body covering of the hexacanth. The very thin cytoplasmic connection between the peripheral layer of tegument and binucleate perikaryon appears only very seldom in the ultrathin sections as a narrow cytoplasmic strand and has a plasma membrane that is reinforced by a single row of cortical microtubules. The HRM covers only one pole of the oncosphere and is attached to the oncosphere surface. The HRM is clearly visible in the mature oncosphere and is draped over the hook blades, the sharp points of which are protected by moderately electron-dense caps. Comparison of the above morphology with that of TEM study of the tegument of adult cestodes shows a great similarity as well as homology in the body covering of both larval and adult cestodes.

## Introduction

The ultrastructure of the oncospheral tegument and hook region membrane cannot be understood without an account of their origin and differentiation (Lee 1966; Lumsden et al. 1974; Threadgold 1984). Detailed TEM studies on oncospheres are very rare because of numerous technical difficulties. So far, they have been conducted on four cestode species: *Hymenolepis diminuta* by Rybicka (1973), *Catenotania pusilla* by Świderski (1968, 1972) and two species of dilepidid cestodes, *Anomotaenia constricta* and *Paricterotaenia porosa* by Gabrion (1981).

Preliminary results on the origin and differentiation of the oncospheral tegument of *Echinococcus multilocularis*, a parasite of medical and veterinary importance, were first obtained about 20 years ago and only published in abstract form by Świderski (1995). The purpose of the present study is a re-examination of the earlier study and re-description of the origin, differentiation and functional ultrastructure of the oncospheral tegument and hook region membrane of *E. multilocularis* with application of new, much improved modern methods of TEM involving cryofixation and TEM cytochemistry.

## Materials and methods

### Materials

Live specimens of *Echinococcus multilocularis* were isolated from the intestine of a naturally infected red fox (*Vulpes vulpes* L.) from La Roche sur Foron (France) captured in June 2014.

### TEM preparation of samples

Adult tapeworms were immediately rinsed with a 0.9% NaCl solution. Later, they were fixed in cold (4 °C) 2.5% glutaraldehyde in a 0.1 M sodium cacodylate buffer at pH 7.4 for a minimum of 2 h, rinsed in 0.1 M sodium cacodylate buffer at pH 7.4, post-fixed in cold (4 °C) 1% osmium tetroxide with 0.9% potassium ferricyanide in the same buffer for 1 h, rinsed in MilliQ water (Millipore Gradient A10), dehydrated in an ethanol series and propylene oxide, embedded in Spurr’s resin and polymerised at 60 °C for 72 h.

Ultrathin sections (60–90 nm thick) of pregravid and gravid proglottids were obtained in a Reichert-Jung Ultracut E ultramicrotome. Sections were placed on 200-μm mesh copper grids and double-stained with uranyl acetate and lead citrate according to the Reynolds (1963) methodology. The grids were examined in a JEOL 1010 transmission electron microscope (Jeol, Japan) operated at 80 kV, in the “Centres Científics i Tecnològics” of the University of Barcelona (CCiTUB).

### Freeze substitution and infiltration with Lowicryl resin

Some specimens were fixed in cold (4 °C) 4% paraformaldehyde + 0.1% glutaraldehyde in a 0.1 M sodium cacodylate buffer at pH 7.4 for 4 to 5 h and then conserved in cold (4 °C) 2% paraformaldehyde in the same buffer. Samples were rinsed in a 0.15 M glycine in a 0.1 M sodium cacodylate buffer at pH 7.4, cryoprotected by crescent concentrations (10, 20 and 30%) of glycerol in the same buffer, and then cryofixed in liquid propane.

Samples were freeze-substituted for 3 days at − 90 °C in anhydrous acetone containing 0.5% uranyl acetate. Then, they were warmed up to − 50 °C, at 5 °C/h (EM AFS2, Leica, Vienna, Austria). After several acetone rinses, samples were infiltrated with Lowicryl HM20 resin for 4 days. Samples were polymerised under UV light: at − 50 °C for 24 h, during the warming up at a rate 5 °C/h until 22 °C, and 48 h at 22 °C.

Ultrathin sections were picked up on Formvar-coated nickel grids, double-stained with uranyl acetate and lead citrate, and examined in a JEOL 1010 TEM operated at 80 kV, in the CCiTUB.

### Cytochemistry

The periodic acid-thiosemicarbazide-silver proteinate (PA-TSC-SP) technique of Thiéry (1967) was applied to determine the localisation of glycogen at the ultrastructural level. Thus, ultrathin sections collected on gold grids were treated as follows: 30 min in 10% PA, rinsed in MilliQ water, 24 h in TCH, rinsed in acetic solutions and MilliQ water, 30 min in 1% SP in the dark, and rinsed in MilliQ water. Gold grids were also examined in a JEOL 1010 TEM operated at an accelerating voltage of 80 kV, in the CCiTUB.

## Results

**Fig. 1:**
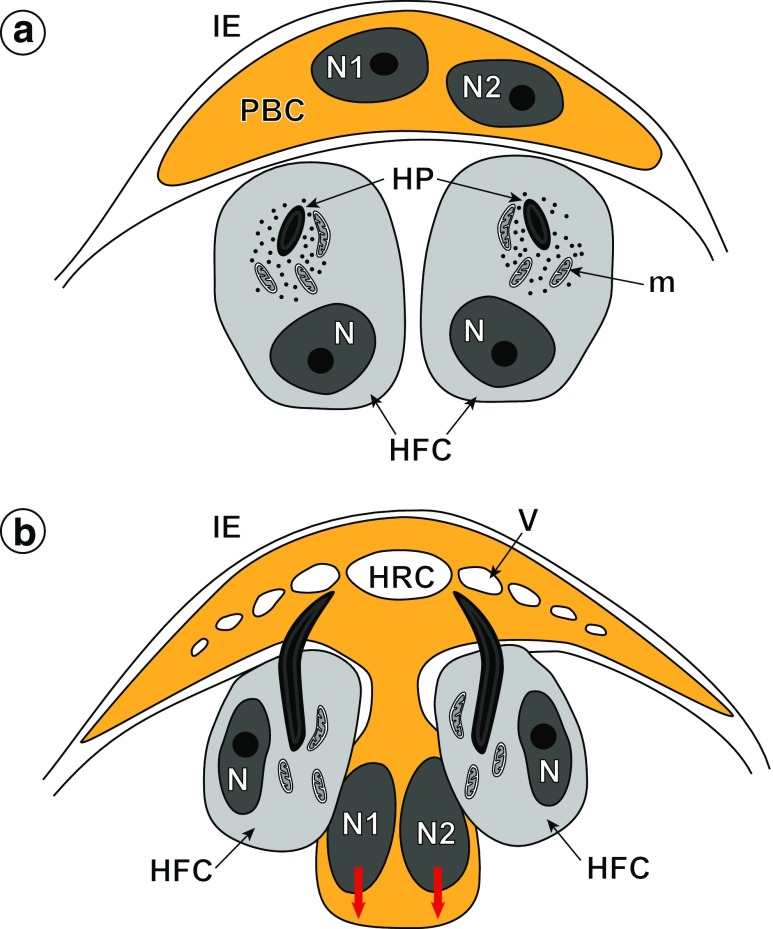
Schematic diagram illustrating the origin (**a**) and beginning of differentiation (**b**) of the oncospheral tegument and hook region membrane in the preoncospheral stage of embryonic development of *Echinococcus multilocularis*. All structures involved in formation of the oncospheral tegument and hook region membrane are marked in orange colour. Two red arrows show direction of progressive sinking or migration of the binucleate subtegumental perikaryon which sunk deep into the central region of differentiating oncosphere. *HFC* hook-forming cell or oncoblast, *HP* hook primordium, *HRC* hook region cavity, *IE* inner envelope, *m* mitochondria, *N1*, *N2* two nuclei of the binucleate complex primordium, *N* nucleus of hook-forming cell, *PBC* binucleate complex primordium, *V* vesicles in the outer cytoplasm, undergoing progressive fusion

**Fig. 2:**
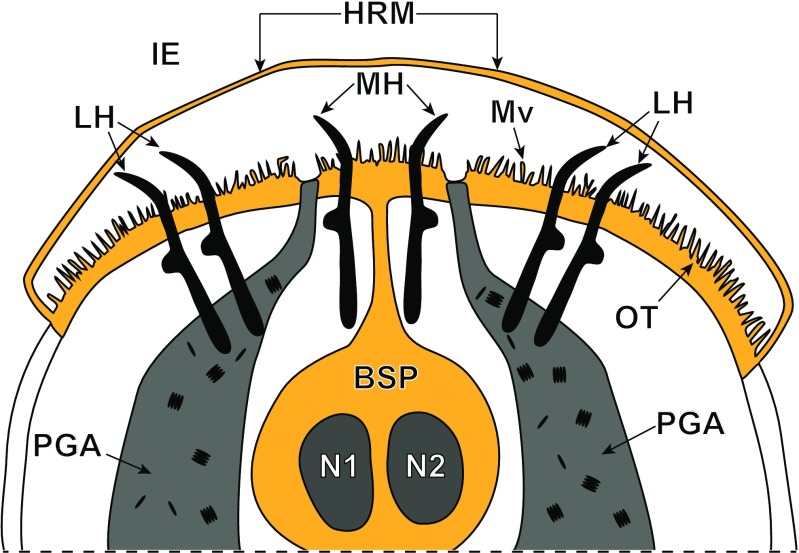
Schematic diagram illustrating the general topography of the oncospheral tegument and hook region membrane in relation to the oncospheral hooks, penetration gland arms and glandular exits in the mature intrauterine eggs. All structures involved in formation of the oncospheral tegument and hook region membrane are marked in orange colour. *BSP* binucleate subtegumental perikaryon, *HRM* hook region membrane, *IE* inner envelope *LH* lateral hooks, *MH* median hooks, *Mv* microvilli, *N1*, *N2* nucleus, *OT* oncospheral tegument, *PGA* penetration glands arms

**Fig. 3:**
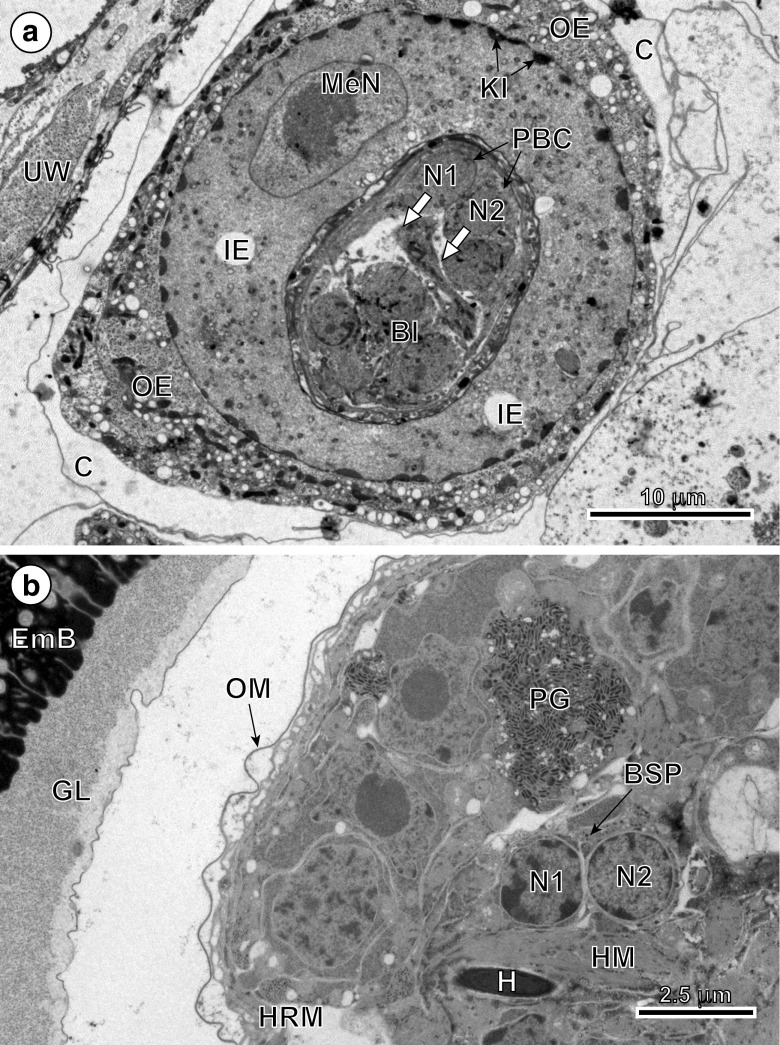
Comparison of an early and advanced preoncospheral stage of embryonic development in *Echinococcus multilocularis*. **a** Ultrastructure of an embryo in the early preoncospheral stage of embryonic development. Note the binucleate complex primordium (PBC), which appears as a syncytial cap or “calotte” situated beneath the inner envelope (IE) at one pole of the developing embryo; two thick arrows mark the direction of progressive migration of both nuclei (N1, N2) surrounded by a thin layer of common cytoplasm and become transformed into the binucleate subtegumental perikaryon. **b** Part of the embryo in the advanced stage of preoncosphere showing the binucleate subtegumental perikaryon (BSP) of the tegumental syncytium sunk deep into the central part of the embryo and surrounded by differentiating blastomeres. *Bl* blastomere, *C* vitelline capsule, *EmB* embryophoric blocks, *GL* granular layer, *H* oncospheral hooks, *HM* hook muscles, *HRM* hook region membrane, *KI* keratin-like protein islands, *MeN* mesomere nucleus, *OE* outer envelope, *OM* oncospheral membrane, *PG* penetration gland, *UW* uterine wall

**Fig. 4:**
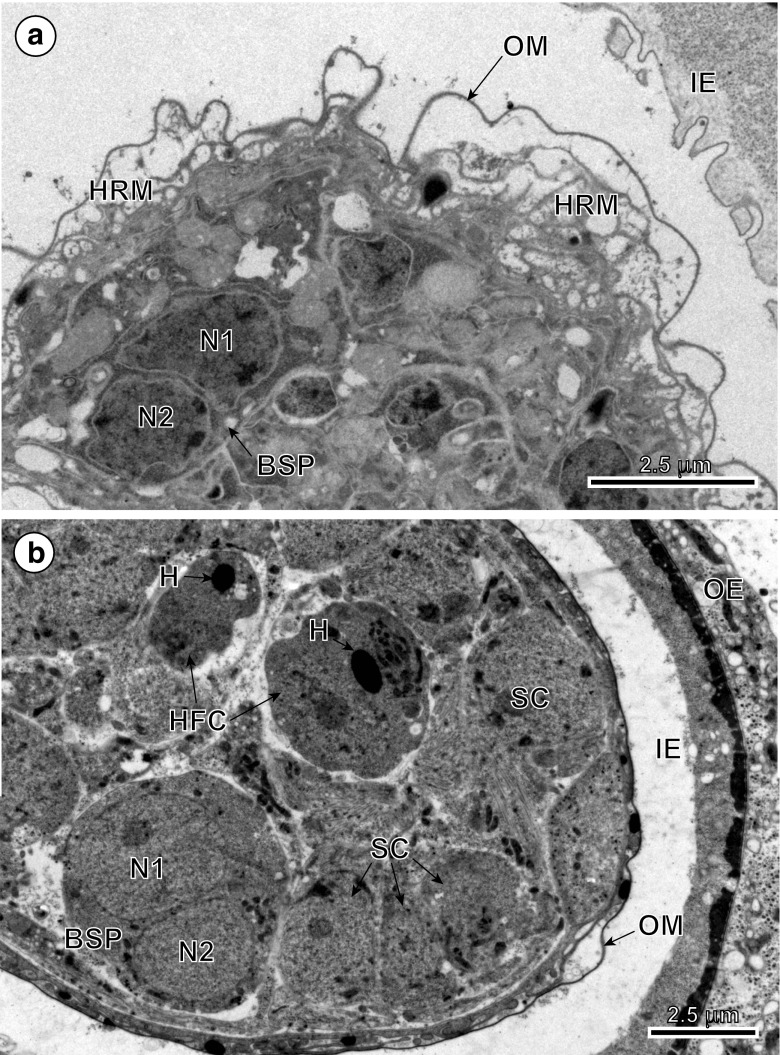
Consecutive stages of a tegumental perikaryon migration. **a** Part of an embryo in the early preoncospheral stage of development showing much infolded oncospheral membrane and the binucleate perikaryon (BSP) of the oncospheral tegument in the early stage of its migration, sunken already below the peripheral layers of oncospheral musculature. **b** Part of the late stage of preoncospheral differentiation showing the binucleate perikaryon of oncospheral tegument sunk deep into the central part of the embryo and surrounded by differentiating blastomeres. *H* oncospheral hooks, *HCF* hook forming cell, *HRM* hook region membrane, *IE* inner envelope, *N1*, *N2*, nucleus, *OE* outer envelope, *OM* oncospheral membrane, *SC* somatic cells

In *Echinococcus multilocularis*, both the oncospheral tegument (OT) and associated hook region membrane (HRM) covering only one pole of the hexacanth, originates from a syncytial binucleate complex (PBC), which appears at the early stage of morphogenesis and organogenesis (Figs. 1a, 2 and 3a). At this stage of the early preoncosphere, the binucleate complex primordium (PBC) appears as a syncytial cap or “calotte” situated beneath the inner envelope (IE) at one pole of the developing embryo (Figs. 1a and 3a). During oncosphere differentiation (Figs. 1b and 3a), the cyton of the syncytium migrates progressively deeper into the central part of the embryo, but remains always connected with the distal cytoplasm by a tendrillar cytoplasmic connection. This connection between the binucleate perikaryon (BSP) and distal, peripheral layer of cytoplasm is very narrow and appears only very seldom in the sections. The binucleate cells of *E. multilocularis* embryos resemble such cells described previously in embryos of other cestode species (for details see Discussion and Rybicka 1973); they have been also identified as the embryonic sunken tegumental cells. The binucleate cells of *E. multilocularis* embryos show all features characteristically associated with differentiating cells, such as large nuclei with prominent nucleoli and numerous dense islands of heterochromatin (HCh) adjacent to the nuclear membrane (Figs. 3b and 4a, b). Their cytoplasm shows abundance of free ribosomes and polyribosomes and sometimes short profiles of endoplasmic reticulum and small membrane-bound granules (Figs. 3b and 4b).

**Fig. 5:**
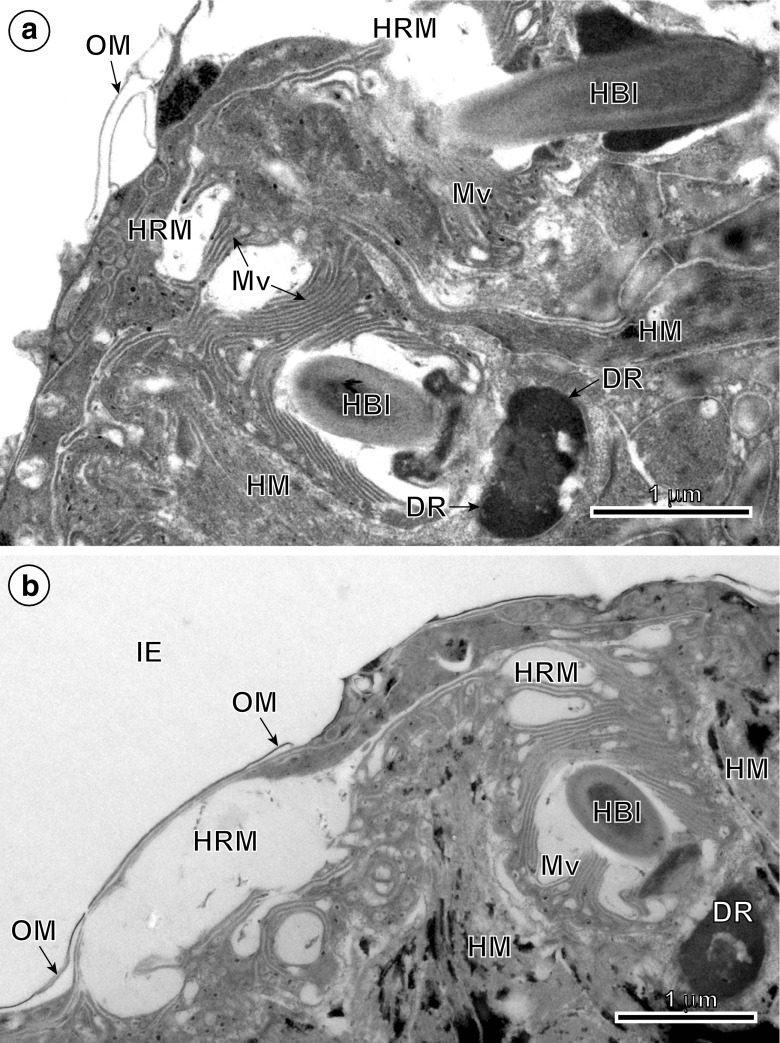
High-power TEM micrographs showing ultrastructural details of the oncospheral tegument and hook region membrane. **a** Note two oblique sections of blades of the oncospheral hooks (HBl), surrounded by numerous long microvilli (Mv) which protrude into large cavity situated under the hook region membrane (HRM) and oncospheral membrane (OM). **b** Oblique section through the hook region membrane showing hook blade exit and oncospheral tegument with numerous long microvilli at its surface. *DR* desmosome rings, *HM* hook musculature, *IE* inner envelope

Following cyton migration, numerous cytoplasmic vesicles (V) appear in the distal cytoplasm. The vesicles join, forming by fusion a single large cavity or lacuna, the walls of which are becoming the walls of two delaminated layers (compare Figs. 2, 4a and 5a, b): the oncospheral tegument distal cytoplasm (OT) and the “hook region membrane” (HRM). This delamination of the initially compact layer of binucleate complex cytoplasm into two layers seems to be closely associated with differentiation of oncospheral hooks, the elongating blades of which protrude progressively into a newly formed cavity (compare Figs. 1b, 2, 3b, 4a and 5a, b). The pressure of hook blades on the hook region membrane appears to facilitate its further separation from the distal cytoplasm. In the mature oncosphere, the surface of the distal cytoplasm forms a regular brush border of cytoplasmic processes or microvilli (Mv). The two main components of the oncospheral tegument (OT) are as follows: (1) the peripheral anucleated cytoplasmic layer with a regular brush border of microvilli (Mv) at its surface and (2) a large binucleate cell of the tegumental perikaryon (BSP) sunk deep into the oncospheral central region; both parts are connected by a narrow cytoplasmic strand (Fig. 2). The distal cytoplasm accumulates numerous membrane-bound, dense granules, mitochondria and vesicles, and represents the true body covering of the hexacanth. The cytoplasmic connection between the distal cytoplasm and the cyton, or “medullary binucleate cell”, appears on longitudinal and cross-sections as a narrow cytoplasmic strand, the plasma membrane of which is reinforced by numerous microtubules.

**Fig. 6:**
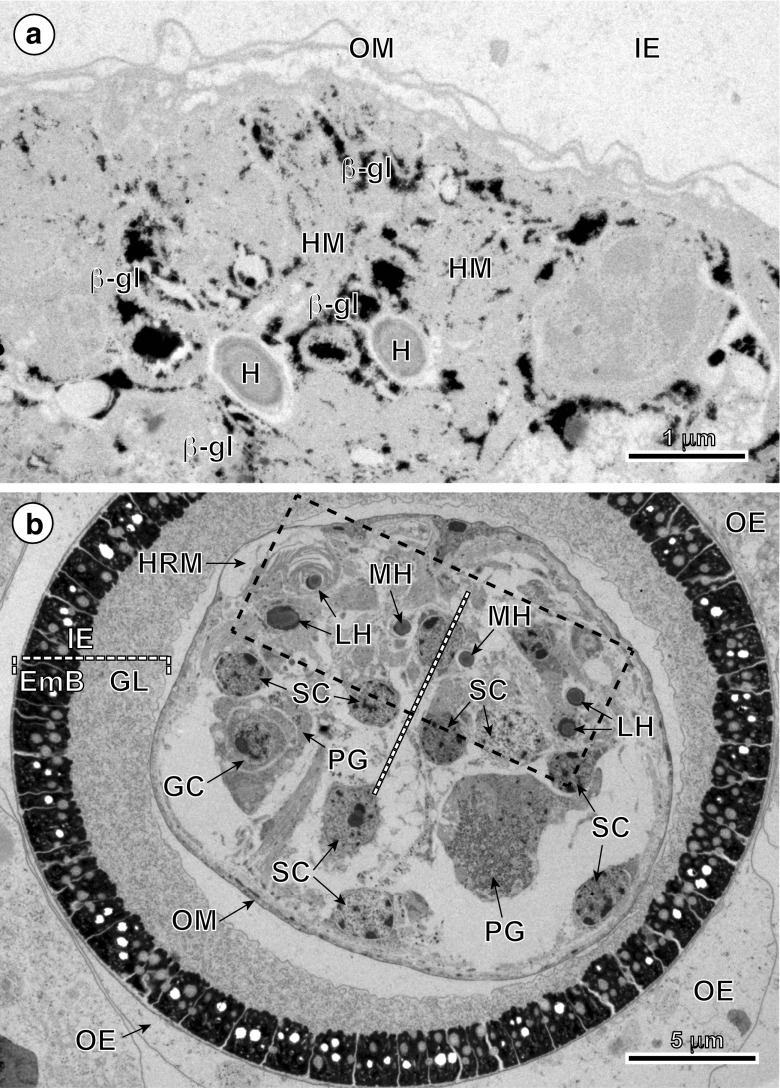
TEM micrographs of mature eggs of *Echinococcus multilocularis*. **a** Part of the oncosphere showing the high concentration of beta-glycogen particles (β-gl) in the musculature of oncospheral hooks (HM) as indicated by the cytochemical test of Thiéry. **b** Low-power electron micrograph illustrating the general topography of mature intrauterine egg. Note: (1) a bilateral symmetry of the oncosphere (white interrupted line) and (2) position of hook region membrane and oncospheral tegument at one pole of the hexacanth. The hook region is marked by a frame composed of interrupted black lines. *EmB* embryophoric blocks, *GC* germinative cells, *GL* granular layer, *HRM* hook region membrane, *IE* inner envelope, *LH* lateral hooks, *MH* median hooks, *OE* outer envelope, *OM* oncospheral membrane, *PG* penetration glands, *SC* somatic cells

The results of the cytochemical test of Thiéry (1967) for detection of glycogen at TEM level are illustrated on Fig. 6a and show that in spite of strongly positive reaction for beta-glycogen particles in hook and somatic musculature there is no trace of glycogen in the oncospheral tegument and HRM.

The general topography of mature intrauterine eggs is illustrated in Fig. 6b. Note: (1) an axis of bilateral symmetry of the oncosphere, marked by a white interrupted line, and (2) position of the hook region membrane and oncospheral tegument at one pole of the hexacanth, marked by a frame composed of interrupted black lines.

## Discussion

The present study indicates that the origin, differentiation and ultrastructure of both the oncospheral tegument and the hook region membrane in *Echinococcus multilocularis* are essentially similar to those reported in the pioneer studies of Rybicka (1973), on *H. diminuta*. At the same time, they strongly confirm the preliminary results obtained by Świderski (1994, 1995) on the same species, but with improved, modern methods of fixation and TEM cytochemistry.

As mentioned briefly in the introduction, numerous technical difficulties limit the number of TEM studies on the development of the oncospheral tegument and hooks. These include problems with proper fixation, embedding and cutting procedures of impermeable, very hard, keratinized structures of cestode eggs, such as different types of egg protective envelopes (e.g. taeniid and anoplocephalid embryophores or oncospheral hooks). As a result, only a very few cestode species have been successfully examined in this respect. Some additional information on this subject can be found in papers describing more general aspects of egg or oncospheral envelope formation in *Catenotania pusilla* by Świderski (1968, 1972), in *Anoplocephaloides dentata* by Świderski et al. (2001a, b), in *Joyeuxiella echinorhyncoides* by Świderski et al. (2004) and *Mosgovoyia ctenoides* by Młocicki et al. (2005, 2006).

The description of the oncospheral tegument in *Hymenolepis diminuta* exhibiting a distal cytoplasm with an internal cyton by Rybicka (1973) suggests homologies between the teguments of the oncosphere, metacestode and adult worm. Rybicka’s observation (Rybicka 1973), confirmed in the present study, is also confirmed by the studies on brush border development in the tegument of the tapeworm *Spirometra mansonoides* by Lumsden et al. (1974), and by numerous more recent studies as reviewed by Threadgold (1984). Presence of binucleate cells in the preoncospheres and oncospheres of different cestode species was frequently reported in several light microscope (LM) studies by Ogren (1956, 1957, 1958), who described them as the “medullary contractile centres”, and reviewed by Rybicka (1966) as well as in the early electron microscopical studies (TEM) by Pence (1970) or Rybicka (1972). In these studies, the interpretation of their function was still highly speculative. It was Rybicka (1973), however, who for the first time identified these binucleate cells as the embryonic sunken tegumental cells. Presence of an oncospheral calotte or hook region membrane (HRM) was described and documented for the first time in the mature oncospheres of *C. pusilla* by Świderski (1972).

The results of Gabrion (1981) on the differentiation of oncospheral tegument and the hook region membrane or “calotte” (his terminology) in *A*. *constrica* and *P. porosa* show similarity with our results only in the initial, earliest stages of calotte differentiation of *E. multilocularis*. In both species of dilepidids, as in *E. multilocularis*, a binucleate cytoplasmic primordium is at the surface of one pole of an early differentiating preoncosphere, just under the inner envelope, as illustrated in Fig. 1 of Gabrion (1981, p. 193). In addition to two nuclei, one of them with a prominent nucleolus, the figure shows also numerous elongated vesicles in different stages of fusion. His TEM micrographs illustrate well the more advanced stages of vesicular fusion, resulting in formation of a large cavity into which the elongating hook blades progressively intrude. During formation and progressive increase in size of the cavity, the initially compact layer of external, anucleated cytoplasm undergoes rapid delamination into the external layer or hook region membrane (HRM), designated “oncospheral calotte” by Gabrion, and the internal/inner layer or oncospheral tegument. A great enigma never elucidated in Gabrion’s paper, however, is the fate of a binucleate cytoplasmic primordium (see p. 193), which becomes anucleated; unfortunately, its fate is never elucidated in his paper.

How does one account for the great differences in the description of the mature embryo and between Fig. 18 of Gabrion (1981, p. 193) and Fig. 2 of the present study that both picture the structure of the cap or calotte? A significant and basic difference is that Gabrion’s figure is incomplete and lacks any sunken, binucleate cyton connected by a very narrow neck to the base of the hook region membrane (HRM). In fact, Gabrion apparently did not find such a connection because it is not indicated anywhere in his description. This omission is perhaps not surprising since we discovered that the tendrillar cytoplasmic connection to the sunken binucleate cyton is “very narrow and appears only very seldom on the sections”. Rybicka (1973), too, found that the connection, “between the binucleate cell and the periphal epithelial layer seldom appeared in sections”. Given that such a connection is not common in sections, it would appear that it simply may not have been present in any of the sections observed by Gabrion, an unfortunate situation exacerbated also by the high magnification—and therefore lower range of field—of his photos. Though Gabrion cited Rybicka’s 1965 and 1972 papers, for some unknown reason, he did not cite her 1973 paper in which the sunken cyton is clearly described. By apparently not having seen the narrow connection of the sunken cyton and contrasted its absence to one described by Rybicka (1973), the fate of the binucleate cell and related events in the latter maturation of the embryo are naturally interpreted quite differently from that described by Rybicka (1973) and the present study. It might also explain why Gabrion (1981, p. 203–204) concluded that the structure of the tegument of the embryo was made up of different cells or “structure en mosaique”, rather than one related to a sunken cyton.

It is interesting to note that the tegumental nuclei in most free-living, non-parasitic Platyhelminthes are localised in the outer, or the most external layer of their tegument (Tyler 1984, 2001), contrary to the situation observed in the parasitic Platyhelminthes where there are tegumental nuclei or tegumental perikarya. The most sensitive cell organelles to the host destructive action, e.g. the effects of host digestive enzymes, therefore always remain sunken very deep in the parenchyma, below several layers of body musculature (Lee 1966; Świderski 1966; Threadgold 1984; Kuperman 1988). Identification of the binucleate subtegumental perikaryon of *H. diminuta* as an embryonic sunken tegumental cell by Rybicka (1973) was confirmed by several more recent reports (Świderski and Eckert 1977; Świderski 2008) as well as by the results of previous, preliminary data (Świderski 1995) and the present results on *E. multilocularis*. All of them shed new light on the origin of the neodermis and tegumental nuclei migration or their sunken nature, observed in the embryonic and different post-embryonic larval stages, and adult parasitic Platyhelminthes. In the case of cyclophyllidean cestodes, the sunken tegumental structure or neodermis is a very important adaptation of these parasites to their parasitic ways of life and is evident in the three stages of their life cycles: hexanths, metacestode stages and adult tapeworms.

The polarity or topographic orientation of the oncosphere has been controversial for a long time, since its interpretation depends upon the criteria used to determine the anterior and posterior pole of the hexacanth embryo; see Mackiewicz (1984) for a review of the polarity orientation of oncospheres. The topographic orientation of the oncosphere is determined by the position of the hooks. According to the conventional scheme, the region opposite, the hook-end is referred to as anterior, i.e. the region of future scolex differentiation, and the hook-end as posterior. On the other hand, since the oncosphere moves and penetrates the villus of its intermediate host’s intestine with the hook region directed forward, this end is therefore considered as anterior. Since this situation leads to much confusion, Ogren (1968, 1971) suggested that there are two polarities in cestode embryogenesis. The primary or somatic polarity occurs during differentiation stages and in infective oncospheres, while the secondary or germinative polarity determines the pattern of larval (post-embryonic) development. The reorientation of oncosphere polarity, from primary to secondary, takes place during larval metamorphosis, following invasion of the intermediate host.
